# The Urinary Microbiome: Role in Bladder Cancer and Treatment

**DOI:** 10.3390/diagnostics12092068

**Published:** 2022-08-26

**Authors:** Veronika Friedrich, Hae Woong Choi

**Affiliations:** Division of Life Sciences, Korea University, Seoul 02841, Korea

**Keywords:** urinary microbiome, bladder cancer, Bacillus Calmette-Guerin therapy, commensal microbes, tumor microenvironment, cytokines, urinary dysbiosis

## Abstract

Commensal microbes have increasingly been found to be involved in the development and progression of cancer. The recent discovery of the urinary microbiome bolstered the notion that microbes might play a role in bladder cancer. Although microbial involvement in bladder neoplastic transformation and metastatic progression, except schisto somiasis, has not been established, accumulating research suggests that dysbiosis of the urinary microbiome can produce a chronically inflammatory urothelial microenvironment and lead to bladder cancer. In this review, we describe how the urinary microbiome might facilitate the development of bladder cancer by altering the host immune system and the kind of cytokines that are directly involved in these responses. We investigated the therapeutic possibilities of modulating the urinary microbiome, including immune checkpoint therapy. The responsiveness of patients to intravesical Bacillus Calmette-Guerin therapy was evaluated with respect to microbiome composition. We conclude by noting that the application of microbes to orchestrate the inflammatory response in the bladder may facilitate the development of treatments for bladder cancer.

## 1. Introduction

Bladder cancer (BC) is the sixth most common cancer in the United States; over 80,000 new cases were recorded in 2020 alone. Worldwide, more than 570,000 patients were diagnosed with BC, and over 200,000 patients died from the disease [1]. BC can be classified into non-muscle-invasive (NMIBC) and muscle-invasive (MIBC) bladder cancer. NMIBC comprises tumors isolated to the urothelium or lamina propria (stages 0 and 1), while MIBC refers to tumors that have invaded the muscle or deeper layers (stages 2–4) [2]. About 70–80% of newly diagnosed patients present with NMIBC, and only 10–20% show progression to MIBC [3]. As with other cancers, a higher tumor stage is associated with decreased survival rates; survival rates for NMIBC are approximately 80–90%, but drop to as low as 15% for MIBC [4]. Thus, depending on the stage, treatment options vary greatly, with intravesical Bacillus Calmette-Guerin (BCG) therapy following transurethral resection of bladder tumors (TURBT) being the standard treatment for intermediate- or high-risk NMIBC patients [5]. Despite these and other treatment options, bladder cancer shows a high recurrence rate. About 70% of patients who do not undergo cystectomy will experience recurrence of the disease [4]. This, combined with a mortality rate that has remained unchanged for over 30 years, has prompted investigations into other potential treatment methods.

The risk of developing BC increases with age, and men are affected nearly four times more frequently than women worldwide [6]. The most common risk factor for BC is smoking, followed by heavy alcohol use and occupational exposure to polycyclic aromatic hydrocarbons or aromatic amines. Chronic inflammation is now considered as an additional risk factor. Chronic inflammation, which is related with BC, can be attributed to host defense mechanisms for microbial infection or cellular injury in reaction to stressors. Schistosomiasis, in particular, is a well-known parasitic infection that can lead to BC [7]. Another player involved in the pathogenesis of BC is the urinary microbiome [8]. The bladder, which was previously thought to be sterile, is now known to harbor its own set of commensal microorganisms [9,10]. Not only does the composition of the urinary microbiome differ between sexes, it also varies between healthy individuals and BC patients [5,11]. Commensal microorganisms in the urinary bladder may be required for maintaining homeostasis and preventing an unwanted inflammatory response. However, disruption in the urinary microbiome can also initiate proinflammatory responses by triggering immune cell infiltration [12], thereby establishing a chronic inflammatory milieu and eventually leading to the development of BC [12,13].

The fact that the urinary microbiome can play a unique carcinogenic role makes it a potential candidate for use as biomarkers in BC [14]. In addition, modifying the urinary microbiome could potentially be used as a therapeutic option in conjunction with conventional treatments [15,16,17]. This review examines the characteristics of the urinary microbiome in individuals with BC, and its capacity to modify the tumor microenvironment (TME) and immune response while promoting or inhibiting BC. In particular, this review explores how the urinary microbiome may contribute to the development of BC by modifying the immune cells of the host. The kind of cytokines directly implicated in chronic inflammation are also described.

## 2. Differences in Urinary Microbiome Sample Collection Methods

Collecting specimens from voided urine is noninvasive and convenient for both clinicians and patients when analyzing the urinary microbiome. However, since the urethra and genital tract are inhabited by a variety of microorganisms, analysis of voided urine samples misrepresents the actual composition of the urinary microbiome. Therefore, additional methods are routinely employed to gather urine samples for analysis. The aforementioned voided urine (sometimes referred to as “midstream urine”) approach is the only non-invasive method and the easiest to perform, making it a popular choice in many clinical studies; however, its disadvantages include contamination and consequent inaccuracy. Placing a catheter transurethrally to collect urine is a more invasive alternative, but it considerably reduces the risk of contamination [18]. Suprapubic aspiration, in which a syringe is injected into the bladder to acquire a urine sample, permits the most precise characterization of the urinary microbiome. This prevents the urine from coming in contact with other tissue areas, minimizing the risk of contamination [19,20]. The primary drawbacks of this method are its high degrees of invasiveness and pain [21]. For accurate characterization of the composition of the urinary microbiome, suprapubic aspiration continues to be the preferred method. Because multiple different factors such as pain and required level of sterility must be taken into consideration, individual patients benefit from a collection method that is appropriate for their unique medical situation. In addition to collecting urine, bladder mucosal tissue samples should also be analyzed to identify the bladder mucosa-associated microbiome [22].

As will be presented later, some bacterial genera are much more abundant in the microbiota isolated from the urine of patients with BC compared to that of healthy patients. Recently, “five suspect genera”, namely *Akkermansia*, *Bacteroides*, *Clostridium* sensu stricto, *Enterobacter*, and *Klebsiella* were found to be over-represented in BC tissue samples when compared to urine samples from the same group of BC patients [23]. This difference implies that these genera directly interact with the bladder mucosal tissue, which suggests that they are potentially associated with the oncogenic transformation process occurring at the bladder mucosa [23]. The bladder-mucosa-related microbiota is distinct from the urinary microbiota and provides the optimal test sample for characterizing the relationship between bacteria and cancer [23]. A more precise characterization of the changes in microbiota composition throughout the progression of BC could offer new potential for developing screening or monitoring tools for BC diagnosis.

## 3. Urinary Microbiome in Healthy Individuals

To comprehend the association between the urinary microbiome and the progression or treatment of BC, the characteristics of a non-diseased, healthy human bladder microbiome must be identified. Compared to other microbiomes found throughout the body, the urinary microbiome has not yet been adequately defined [24]. The few clinical trials that have been conducted typically suffer from constraints such as small sample size, imprecise method of urine collection, and high heterogeneity in characteristics such as race and age [25]. Defining a single general set of bacteria that constitutes a healthy bladder is thus challenging, but comparative studies have shed some light on overlapping and diverging urinary microbiome compositions. In terms of microbiomes of other organs, the gut shows different microbiome compositions depending on the sex of the individual [26,27]. Considering the substantial anatomical and physiological disparities between the lower urinary tract of men and women, the urinary microbiome of the bladder unsurprisingly shows differential stratification as well (Table 1) [9,10]. One study found that the most prevalent phyla shared among men and women were *Firmicutes,* followed by *Actinobacteria, Bacteroidetes,* and *Proteobacteria*. In terms of genera, however, the two diverge; healthy females show an abundance of *Streptococcus, Lactobacillus,* and *Prevotella*, while males have greater numbers of *Lactobacillus, Corynebacterium,* and *Gardnerella* [28]. Yet another study characterized healthy male bladders by an abundance of *Enterococcus*, *Proteus,* and *Klebsiella*, and healthy female bladders show dominance of *Lactobacillus* [10]. An independent study also confirmed the abundance of *Lactobacillus, Prevotella,* and *Gardnerella* in the female microbiome [29]. Even though *Lactobacillus* seems to be associated with a healthy urinary microbiome in women in particular, not all strains of the genus seem to be entirely favorable; Pearce et al., reported that the strain *L. gasseri* was more often found in women with urinary urge incontinence, while *L. crispatus* was more frequently cultured in healthy control subjects [30]. Acid-producing *Lactobacillus* species can contribute to keeping pathogenic bacteria unable to survive in a more acidic environment at bay, and thus assume a protective role in the bladder [10]. Indeed, a recent study found that *Lactobacillus* in the bladder can protect the host from uropathogenic *E. coli* infection by triggering type I IFN production [31]. Price et al. found that *Lactobacillus* in women showed no correlation with various factors such as age but discovered that other genera do differ among age groups; *Gardnerella* and *Escherichia* were more commonly found in younger and older women, respectively [32]. Both studies noted that genera composition is highly variable between individuals, once again substantiating the fact that a single, clear definition of what comprises a healthy urinary microbiome is difficult to provide [28,32]. 

Table 1 provides an overview of selected studies that have attempted to characterize the urinary microbiome of healthy males and females. Notice the inconsistent methods of urine collection, in addition to the variability in subject gender, race, and age. Overall, we see a dominance of *Lactobacillus, Prevotella,* and *Streptococcus*. Further studies, ideally separately for different age groups, races, and genders, should be performed to more closely discern which members of the urinary microbiome are consistently found in the bladders of healthy individuals.

## 4. Urinary Microbiome in Bladder Cancer Patients

Urinary dysbiosis can be defined as the loss of beneficial bacteria from a healthy urinary bacterial community [37]. As was previously described, determining commensal microbes as “beneficial” is challenging; additionally, several studies have reported interpersonal variation even among healthy patients [8,24,37]. Recently, dysbiosis of the urinary microbiome has been linked to pathological conditions such as urinary urge incontinence, interstitial cystitis, and overactive bladder [10,29,30,38]. Although a similar speculation was made for BC patients, it is still unknown whether dysbiosis of the urinary microbiome at baseline precedes and is a risk factor for BC, or whether BC is responsible for the alteration of the microbiome. To date, few studies analyzing the urinary microbiome in bladder cancer have been performed, limiting our ability to draw definitive conclusions. A summary of the results from the available studies is given in Table 2.

*Streptococcus*, *Veillonella*, and *Corynebacterium* were found to be abundant in patients with BC, but no differences were observed in alpha diversity compared to healthy individuals [8]. A subsequent study confirmed that only beta diversity is elevated in cancerous bladder samples [41]. Other independent studies reported that the genera *Brucellaceae, Acinetobacter,* and *E.Shigella* were commonly present in BC patients; however, bacterial richness and diversity were significantly reduced in their urinary microbiome [22,40]. 

In contrast to the relationship between the urinary microbiome and BC, the bacterial diversity in BC increases with an increase in the genera *Acinetobacter*, *Anaerococcus*, *Rubrobacter*, *Staphylococcus*, *Streptococcus*, and *Prevotella* [14,39]. The genus *Acinetobacter*, the incidence of which was found to be elevated in BC specimens, has also been reported to contribute to the development of multidrug resistance and cause a variety of diseases, including pneumonia and bloodstream infections [43,44]. Multiple studies demonstrate an increase in the phylum *Proteobacteria* in BC tissues. Other studies examining gut pathogenesis also found that dysbiosis of genera within this phylum are correlated with Crohn’s disease and colitis-associated colorectal cancer [45,46]. Therefore, an increase of *Acinetobacter*, and more broadly *Proteobacteria*, could be employed as dysbiosis markers to diagnose BC. Oresta et al. noted that *Ruminococcus* and *Bifidobacterium*, which were reduced in BC patients, are genera known to be anti-inflammatory and important in mucosal homeostasis [42,47,48]. A decrease in beneficial bacteria such as these may provide “bad” bacteria with a permissive environment to stimulate inflammation and oxidative stress. They also suggest that tumor and microbiome may interact, where tumor formation breaks down the normal urothelium and favors attachment and proliferation of certain taxa [42]. As mentioned before, the development and progression of BC is thought to be influenced by many factors, which seems to result in a differential incidence and mortality rate between men and women. Even though the incidence is higher in men, poorer outcomes and higher death rates are associated with BC in women [49,50]. Studies have implicated hormones as playing an important role in BC, and more recently, sex-associated differences in the urinary microbiome have been emphasized [50,51]. Just as in healthy subjects, the urinary microbiome thus also shows variation between male and female bladder cancer patients: while the genera *Pelomonas, Corynebacterium,* and *Finegoldia* are more abundant in men with BC, women with BC show higher levels of *Lactobacillus, Actinotignum,* and *Prevotella* [41]. Oresta et al. found male BC patients to show an abundance of *Veillonella* and *Corynebacterium*, while Wu et al. reported an abundance of *Acinetobacter*, *Anaerococcus*, and *Rubrobacter* [14,42]. Although sex differences in the urinary microbiome were observed in recent studies, a more extensive mechanistic investigation is required to establish a link between these differences, the higher incidence of BC in men and worse disease progression in women.

Mechanically, urinary microbiota, which can attach to bladder mucosal surfaces, can undertake continuous translocations or transient tissue invasions [12]. Furthermore, bacteria present in the bladder can create biofilms that allow for continuous and extended direct contact with the urothelium, although it is unclear whether urinary microbiota can form biofilms in the bladder [12]. Due to the fact that a variety of bacteria create proteases that can act intracellularly and/or extracellularly, perturbing the natural process of extracellular matrix (ECM) renewal in the bladder can result in an altered and potentially cancer-promoting extracellular milieu [12,52]. It has been found that *Pseudomonas aeruginosa* secretes alkaline proteases predominately when cultivated in anaerobic conditions (probably within bladder tumor masses) [12]. These alkaline proteases degrade components of the ECM and inhibit lymphocyte proliferation by degrading and inactivating IFN-γ [53]. Additionally, proteases can cleave the IL-6 receptor, the FAS ligand, TNF, and its receptors [52,54,55]. Therefore, urinary microbiota and their released proteases may influence the integrity of the urothelial barrier, ECM development, and immunological repertoire in the bladder. This altered bladder tissue can thus potentially facilitate the development of the bladder tumor microenvironment.

The studies cited in this review show only a small number of overlapping genera and high variability even within a single study. Moreover, there is a substantial degree of interindividual variability in the microbial composition of both cancer patients and healthy individuals [8,40]. As was described in the previous section, the method of sample collection is a major factor determining the disparate results of urinary microbiome investigations. Some of the studies were conducted with people of a single sex, while others included participants of both sexes. Additional characteristics, such as geographic location, age, and race, play a significant influence in the difficulty of identifying bacterial genera that are related to BC globally.

## 5. Tumor Microenvironment in Bladder Cancer

Before exploring the relationship between the urinary microbiome and TME, as well as their therapeutic applications, one first has to understand the characteristics of the TME in BC. When it comes to the treatment of BC, BCG remains the gold standard therapy for NMIBC decades after its first application, with the treatment of locally advanced or MIBC having undergone quite a few changes. There have also been recent advances in immune checkpoint inhibitors in the treatment of BC: Immunotherapy in the form of the PD-1 inhibitor, pembrolizumab, has been approved for high-risk NMIBC patients who did not respond to BCG treatment [56]. Because immunotherapy acts on the TME and is becoming a more popular and highly researched topic in the context of BC, the composition of the TME and the contribution of each component must be understood. Broadly speaking, the actions of every player involved in the TME can be classified as being either inflammatory or immunosuppressive. Even though inflammation is usually a positive sign of an ongoing immune response, a microenvironment that is chronically inflammatory is also essential for the initiation, progression, and metastasis of tumors [57]. Additionally, immunosuppression is just as important for the survival of cancer cells. As the tumor stage advances, immunosuppressive mechanisms utilized by cancer cells create an environment that prevents the immune system from working properly, thereby affecting the efficacy of immunotherapy [58,59]. In the following section, we provide an overview of the involvement of different immune cell populations in the development of the TME in BC.

### 5.1. Macrophages

A hallmark of the TME of neoplasms is the presence of tumor-associated macrophages (TAMs). TAMs play critical roles in the promotion of metastasis and maintenance of inflammation. Because activated macrophages can be polarized into the M1 or M2 type, BC employs both to evade the immune response and metastasize. While M1 macrophages are considered to be pro-inflammatory, M2 macrophages represent the immunosuppressive phenotype [60]. M1 macrophages, through the release of proinflammatory cytokines IL-23, IL-17, and IL-6, can drive tumor growth [60,61,62]. In addition, this subtype promotes the vascularization of tumor cells by releasing VEGF and MMP9, and is also involved in the epithelial-mesenchymal transition (EMT), pushing the invasion of tumor cells into benign tissue [60,63]. Even more useful to tumors are macrophages of the M2 type, which produce immunosuppressive cytokines, such as IL-4, IL-10, IL-13, or TGF-β. These secreted cytokines impede the anti-tumor immune responses and favor cancer cell proliferation, invasion, and metastasis [64,65]. TAMs, which exhibit the M2 phenotype, have been shown to be recruited by CCL2 in bladder cancer, promoting lymphatic metastasis via VEGF-C secretion [66]. In addition, higher TAM/TIL (tumor infiltrating lymphocyte) ratios showed strong association with poor survival outcomes in MIBC [67]. Recently, M2 macrophages were confirmed to dominate in BC and were correlated to aggressiveness and poorer overall survival [68,69,70,71]. Sustained TAM or M2 infiltration has also been linked to significantly decreased efficacy of BCG treatment [64,72,73,74].

### 5.2. Myeloid-Derived Suppressor Cells

Myeloid-derived suppressor cells (MDSCs) are a heterogeneous population of cells consisting of myeloid progenitor cells and immature myeloid cells that can suppress T cell anti-tumor responses [75,76,77]. BC tissue shows higher numbers of MDSCs compared to normal tissue, and this is associated with size, stage, and pathological tumor grade [75,78,79]. Bladder cancer cells produce prostaglandin E2 (PGE2) to activate myeloid progenitor cell differentiation to MDSCs and induce them to express PD-L1 [80,81]. Additionally, bladder cancer cells or BCG treatment recruit and activate type 2 innate lymphoid cell (ILC2) to produce IL-13 that can amplify MDSC recruitment and induction via CXCL2/MIF-CXCR2 signaling [82,83]. Recruited MDSCs have been associated with progression and tumor-induced immune dysfunction [75,84,85,86,87]. Mechanistically, the enrichment with suppressive molecules such as Arg1, iNOS, ROS, PDL-1, and P-STAT3 enabled MDSCs in BC tissues to effectively suppress T-cell proliferation [83]. A clinical study of urothelial carcinoma patients found elevated numbers of MDSCs that were indeed able to interfere with proliferation and IFN-γ production of T cells [88]. Yang et al. confirmed that higher quantities of MDSCs were present in BC patients and were correlated with stage and poor prognosis [75]. Therefore, MDSCs can be a good diagnostic marker for bladder cancer progression and a cellular target for suppressing BC.

### 5.3. Tregs

Foxp3^+^ regulatory T cells (Tregs) are a subset of CD4^+^ T cells that originate in the thymus or are activated locally by TGFβ, IL-10, or IL-2, and are implicated in the TME of numerous cancers [89,90]. Due to their propensity to inhibit antitumor responses, Tregs play a significant role in the development of an immunosuppressive environment that is advantageous to BC [91]. In NMIBC, increased Treg counts were related with higher tumor grade and significantly correlated with TAM counts [92]. The establishment of a tumor favorable microenvironment mediated by Tregs and TAMs explains the poor response to intravesical BCG immunotherapy [92]. High percentages of Foxp3^+^ Tregs were detected in the peritumor tissue of NMIBC, and a high recurrence rate following TURBT was strongly correlated with the presence of peritumor Tregs [93]. A particular Treg subtype was identified in MIBC. A high frequency of CCR8^+^ Tregs in patients with MIBC was associated with poor prognosis and an immunologic tolerance mediated by CCR8^+^ Tregs [94]. The significance of the CCR8-mediated antitumor response was further supported by the fact that blocking CCR8 could reactivate the antitumoral immune response and enhance sensitivity to anti-PD-1 therapy [94]. Mechanistically, extensive S1P1 expression in bladder cancer tissues was positively correlated with the number of tumor-infiltrating Tregs, which was mediated by the increased expression of TGF-β and IL-10 [95]. It can be concluded that Tregs serve a crucial role in inhibiting the anti-tumor activities of the immune system and are, therefore, risk factors for BC.

### 5.4. Mast Cells

Mast cells are well-known for their central role in allergies and have been linked to numerous types of cancers; however, research on mast cells in BC is limited. High numbers of tumor-infiltrating mast cells in the bladder stromal tissue was an unfavorable prognostic indicator for MIBC patients, whereas patients having low stromal mast cell numbers exhibited a better response to adjuvant chemotherapy [96]. A correlation was observed between an increased number of mast cells and transitional cell carcinoma in urinary bladder tumors, as well as tumor angiogenesis, which contributes to the development of higher-grade BC tumors [97,98,99].

Mast cells store a range of pre-generated mediators such as proteases, histamines, and serotonin, and secrete newly synthesized cytokines and chemokines. Mechanistically, when these mast cells migrate to tumors, they augment the interaction between estrogen receptors (ERs) and C–C Motif Chemokine Ligand 2 (CCL2), with CCL2 promoting EMT and matrix metalloproteinase (MMP) synthesis at the tumor site [100]. This implies that the stimulation of the ER/CCL2/EMT/MMP axis by mast cells enhances the invasion of BC cells [100]. C-Kit positive mast cells are also able to increase microvessel density by promoting angiogenesis, contributing to tumor growth and invasion [98]. This pro-tumor function of mast cells was further supported by the finding that high-risk groups with shorter survival periods possessed a greater number of mast cells [101].

Contrary to the pro-tumor role of mast cells in BC, clinical research showed that few patients with carcinoma who received BCG therapy exhibited an increase in the number of IL-17-positive MCs. The presence of more IL-17^+^ mast cells was associated with a longer period of time without recurrence [102]. Therefore, IL-17^+^ mast cells could be considered a unique mechanism for BCG immunotherapy in BC [102]. Higher expression of CXCL12 in individuals with BC was associated with mast cells in resting state and longer survival [103]. Altogether, as one of the first cells to enter tumors, mast cells are vital immune cells that can cause angiogenesis and promote tumor growth. In contrast, mast cells are also known to be advantageous for patients because they specifically recruit immune cells to the tumor site. Their conflicting contribution to the growth and development of BC requires further research.

## 6. Microbiome-Mediated Cytokines for Tumor Microenvironment Establishment

Microbiota and cancer formation have long been associated, both directly and indirectly. The possibility that microbial dysbiosis is an oncogenic factor in bladder squamous cell carcinoma was bolstered by the observation that *Schistosoma* infection might stimulate the synthesis of N-nitrosamine [7,104]. If urinary microbiota are implicated in bladder cancer and are modulating anticancer drug efficacy, one also needs to investigate the pivotal inflammatory cytokines involved in this process. Numerous cytokines such as IL-6, IL-10, IL-23, TNF-α, and TGF-β were found to be associated with carcinogenesis and the development of cancers, functioning as pro- or anti-tumorigenic [105,106]. Chronic inflammation of the bladder, as observed in schistosomiasis, and long-term catheter use is a well-recognized risk factor for BC, and inflammation associated with superficial BC is a frequent finding during surgery [107,108]. Identifying potential tumor markers will aid in stratifying and determining the malignant potential of a tumor and consequently, response to various therapies. We present an overview of which inflammatory cytokines are influenced by the microbiota and how these interactions contribute to the development of cancer.

### 6.1. IL6

As a pleiotropic cytokine with diverse systemic roles, IL-6 plays a crucial role in inflammatory processes, including immunological diseases and cancers. Specifically, IL-6 has been described as a prognostic factor in cancer. IL-6 was overexpressed in specimens of BC compared to non-cancerous tissues [109]. Positive IL-6 staining was strongly associated with a more advanced clinical stage, a higher recurrence rate following curative treatment, and a lower survival rate [109,110,111]. Patients treated with radical cystectomy (RC) for BC, who had elevated pretreatment plasma levels of IL6 and IL6 receptor (IL6sR), showed poorer oncological disease survival [107]. The IL-6-174G>C polymorphism was significantly associated with BC risk [112,113]. Therapeutically, IL-6 inhibition diminished both tumor development and invasiveness by decreasing cell proliferation, EMT, DNA methyltransferase 1 expression, and angiogenesis [109,114]. Therefore, IL-6 may be a significant predictor of the clinical stage and prognosis of BC, and inhibiting IL-6 may be an effective treatment strategy [109].

IL-6 levels are associated with elevated numbers and increased proliferation of MDSCs in MIBC tissue [115]. IL-6-stimulated MDSCs can severely inhibit the proliferative capacity of CD4^+^ or CD8^+^ T cells [115]. Therefore, IL-6-stimulated MDSCs could support the progression of MIBC by not only speeding up the proliferation of BC cells, but also enhancing their immune suppressive capacity by activating the MAPK signaling pathway [115]. IL-6 induced the phosphorylation of Signal Transducer and Activator of Transcription 3 (STAT3) at Y705, a transcription factor that is elevated in invading basal-type BC compared to luminal BC [116]. As was shown by their co-localization in pSTAT3^+^ BC cancer cells, MYC and FOSL1 are important STAT3 downstream targets. The BBN-induced mouse model of basal-type BC replicated these findings to a significant extent [116]. Cancer-associated fibroblasts (CAFs) expressed significant levels of the IL-6 cytokine, while RT4 bladder cancer cells contained the IL-6R receptor. Culturing RT4 bladder cancer cells with CM iCAF significantly enhanced cell proliferation, migration, and invasion [117]. The suppression of CAFs-secreted IL-6 with a neutralizing antibody effectively reversed the IL-6-induced EMT phenotype, indicating that this cytokine is required for CAF-induced EMT in the evolution of human BC [117]. 

The Anti-IL-6 m Ab therapy, which reduced the incidence of anorexia and cachexia associated with cancer, may also be effective in the treatment of cancer patients [118]. Microbiota application has been presented as an alternate method for reducing IL-6-mediated responses. Six mixed microbial strains—*Streptococcus thermophilus*, *Lactobacillus rhamnosus*, *Lactobacillus acidophilus*, *Lactobacillus casei*, *Bifidobacterium bifidum*, and *Bifidobacterium longum* were found to be effective in inhibiting IL-6 secretion from peripheral blood mononuclear cells (PBMCs) exposed to LPS [118]. Mechanistically, the microbiota metabolite butyrate reduced the LPS-induced production of IL-6 from mucosal macrophages. In particular, these mucosal macrophages suppressed the production of Il6 and Nos2 mRNA through transcriptional inhibition [119,120]. In BC, further research is required to determine the effect of microbiota on IL6 expression. In conclusion, the evidence demonstrates that IL-6 is a critical participant in the inflammatory process of cancers, the microbiota may also play a role in the pathway in conjunction with IL-6.

### 6.2. Tumor Necrosis Factor

Tumor necrosis factor (TNF) plays a crucial function in inflammation and has been termed the “master regulator” of inflammatory cytokine production. Therefore, anti-TNF medication has been extensively used to treat a variety of inflammatory illnesses [121]. In addition to inflammation, TNF is also linked to carcinogenesis, especially leading to the progression of BC. TNF-α promotes tumor formation by modulating the release of metalloproteinase and possesses angiogenic activities, which may be essential for cancer invasion and metastasis. Due to the increase in H_2_O_2_, the release of TNF-α during inflammation is connected with the transformation of BC [122]. The serum level of TNF-α is significantly elevated in BC patients with or without schistosomiasis infection; moreover, TNF-α levels are significantly higher in T3 and T4 advanced-stage patients than in T1 and T2 early-stage patients, suggesting that TNF-α level may contribute to the progression of BC [123]. Higher TNF-α expression was found in tumor tissue compared to healthy urothelium, and this elevation was connected to tumor size, recurrence, and progression in grade and stage, which was exerted by the angiogenic effect of TNF-α [124]. TNF-α is also implicated in enhancing invasion and migration of bladder cancer cells by inducing matrix metalloproteinase-9 (MMP-9) release in the TME [125].

Although TNF-α itself alters the TME, TNF gene single nucleotide polymorphisms appear to be associated with BC susceptibility. A genetic relationship between the TNF polymorphisms and the risk of BC and tumor grade at presentation was observed [126]. However, TNF polymorphisms were not correlated with the recurrence or advancement of the disease [126]. Well-known mechanisms potentially link polymorphisms that upregulate inflammatory pathways to cancer, including the production of free radicals and DNA damage, increased angiogenesis, and immunosuppressive effects at higher concentrations [127,128].

Recent studies reported that TNF-α secretion in cancer patients is associated with the signatures of specific microbiota. The presence of commensal bacteria can regulate TNF-α secretion from inflamed tissue. Dysbiosis mediated by specific pathogenic strains might affect the epithelial secretion of TNF-α and cause inflammatory responses in tissues [129]. This has been supported by the fact that when compared to controls, epithelial tumor *TNF-α* mRNA expression and inflammatory mediators were observed in germ-free IL10^−/−^ plus *Klebsiella pneumoniae* mouse model [129]. Increased TNF-α secretion caused by *Fusobacterium nucleatum* infection modulates the expression levels of the cancer-carcinogenic target MiR-21, which in turn modulates the secretion of additional inflammatory cytokines, including TNF-α, IL-6, IL-17A, and IL-21 [130,131,132].

Commensal probiotic bacteria can decrease the expression of TNF-α, resulting in an anti-inflammatory effect. Numerous studies have examined the association between TNF, microbiota, and colorectal cancer. *Lactobacillus rhamnosus* GG and its secreted components exhibited anti-inflammatory and anti-apoptotic actions in colon epithelial cells by reducing TNF-α-induced apoptosis in HT-29 human colorectal cancer cells and ameliorating TNF-α-induced epithelial damage in mouse colon explants [133]. In a mouse model of colitis-associated colorectal cancer, a membrane protein isolated from *Akkermansia muciniphila* delayed tumor growth and decreased tumor number and size by boosting CD8^+^ T cells and TNF- production in the colon [134].

Because the NF-κB cascade is a central link in the communication within host-microbial interactions during the occurrence and development of BC, it induces an inflammatory microenvironment by promoting the production of pro-inflammatory cytokines, containing TNF-α, IL-1β, IL-6, and IL-8 [135,136]. An epidemiologic study of bladder carcinoma patients from ten geographic areas of the United States demonstrated that urinary tract infections were strongly related to squamous cell carcinoma [137]. Although limited information is available to identify the role of urinary microbiome in BC, dysbiosis mediated by urinary tract infection, which is a strong source of TNF-α secretion, may play a critical role in the onset of BC.

### 6.3. Interleukin-17

The IL-17 family consists of pro-inflammatory cytokines IL-17A–F, which are mostly released by Th17 cells [138]. Because IL-17 and the IL17 receptor have been examined and licensed for use in patients with a variety of inflammatory disorders, including psoriasis, psoriatic arthritis, and ankylosing spondylitis, it will be interesting to examine their influence on BC. IL-17 is related to all tumorigenic processes, including tumor development, proliferation, and angiogenesis [139]. The detection of IL-23R and IL-17 levels has clinical relevance in the diagnosis and prognosis of BC [140]. The expression levels of IL-23R and IL-17 were elevated in the tumor tissue and serum of patients with BC, and these expression levels were associated with a poor prognosis [140]. IL-17 may be a prognostic tumor marker: In a study, the group with low IL-17 had a greater survival rate and lower microvessel density in the tumor tissue compared to the group with high IL-17 [141]. Therefore, IL-17 has emerged as a potential therapeutic target for metastatic cancer due to its ability to inhibit pathologic angiogenesis [142]. Deregulation of the IL-23/IL-17 pathway is associated with bladder urothelial carcinoma risk because IL-23R is a critical component of the T-helper 17 cell-mediated inflammatory process that orchestrates inflammation, thereby promoting tumor growth [143]. Higher expression levels of IL-23R and IL-17 in tumor tissue and serum were correlated with shortened overall- and disease-free survival, and growth of bladder carcinoma was reduced in IL-17 knockout mice [140,144,145]. Specifically, the rs-1884444 G/T variant of IL-23R was strongly linked to a decreased risk of bladder urothelial carcinoma [143]. 

Similar to other cytokines that have an effect on BC, the association between the urinary microbiome and IL-17 in BC has been examined. By discussing the significance of the gut microbiome in colorectal cancer, we can speculate on the relevance of the urinary microbiome in bladder cancer. *Bacteroides fragilis* is a prevalent commensal in the gastrointestinal tract, and enterotoxigenic *Bacteroides fragilis* was more prevalent in colorectal cancer patients than in healthy controls [146,147,148]. This study demonstrated that enterotoxigenic *Bacteroides fragilis* stimulated the growth of tumor tissue in a way dependent on IL-17 [146,147,148]. Multiple myeloma progression appears to be accelerated by a paracrine signaling network between adaptive and innate immunity that is triggered by commensal bacteria. *Prevotella heparinolytica* stimulates the development of Th17 cells invading the gastrointestinal tract and moving to the bone marrow of transgenic mice, where they accelerate the advancement of multiple myeloma [149]. Consistent with the studies performed on mice, clinical results showed that higher levels of BM IL-17 predicted faster disease progression in multiple myeloma [149].

**Figure 1:**
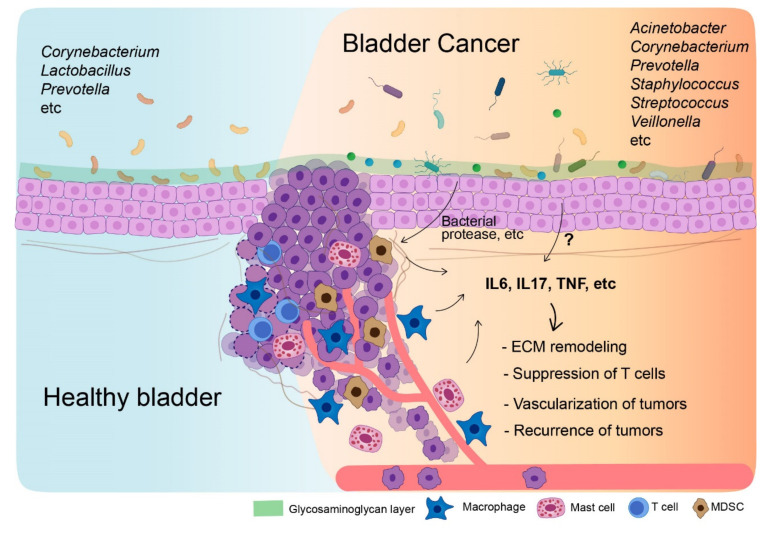
The interaction of urinary microbiota with bladder cancer cells and immune cells. The illustration depicts the difference in microbiome between a healthy bladder and a bladder affected by cancer. Microbiota causing dysbiosis in bladder cancer interact directly or indirectly with immune cells by inducing the secretion of various cytokines. These cytokines accelerate the progression of bladder cancer.

By secreting propionate, microbiota-derived compounds, gut microbiota inhibits IL-17 production by cecal γδT cells [150]. Reduced synthesis of short-chain fatty acids is a consequence of the effect of vancomycin on the gut microbiome. Propionate from gut microbiota reduced intestinal γδT cell production of IL-17 and IL-22 and blocked their synthesis of IL-17 in a histone deacetylase-dependent manner [150]. Altogether, based on studies of gut microbiota elucidating its role in determining the progression of colorectal cancer, the urinary microbiome may also regulate IL-17 and influence the progression of BC. Further studies are required to regulate IL-17 by utilizing the urinary microbiome and use this strategy as biomarkers or therapeutic targets. Lastly, Figure 1 describes the microbiome-immune-cancer axis that occurs in bladder cancer.

## 7. Urinary Microbiome and BCG Responsiveness

Intravesical immunotherapy using BCG is the gold standard adjuvant therapy for high-risk NMIBC and an option to treat intermediate-risk NMIBC [2]. Following TURBT, in the induction phase of the therapy, BCG is instilled through a catheter into the bladder for 90 to 120 min once a week for 6 weeks. Patients with intermediate-risk tumors continue receiving therapy for 1 year, while high-risk tumors require a treatment length of 1–3 years [2,151]. When successful, BCG improved the recurrence-free rate from 26% to 47% [152]; however certain adverse effects including cystitis, bacterial infection, and malaise are common [153]. More severe, albeit rare, side effects include prostatitis, mycobacterial pneumonia, and local complications of urethral obstruction and bladder contracture [154]. Even though this therapeutic approach has been in use for over 40 years, its exact mechanisms are complex and not fully elucidated, leading to complications in efforts to improve efficacy and prevent side effects.

BCG instillation is followed by attachment to urothelial cells through the interaction of fibronectin on the urothelium and the fibronectin attachment protein (FAP) on BCG [155]. The degree to which BCG is internalized by BC cell lines varies, but it seems that BC cells initiate the uptake via micropinocytosis dependent on Rac1 and Cdc42 [156,157]. This internalization is closely linked to a deletion in *PTEN* or mutation in *RAS* oncogenes, and thus, cells that are weakly BCG-reactive can be stimulated by *PTEN* knockdown or oncogenic *RAS* induction to increase BCG uptake [157,158,159]. BCG therapeutic success possibly depending on the presence of certain mutations in carcinoma cells could offer an explanation as to why some patients present with BCG refractory bladder cancer. Furthermore, since instillations begin after TURBT, the majority of BCG interactions should be formed with benign urothelial cells, and more studies need to be performed to scrutinize the exact mechanisms in vivo [159]. 

BCG therapy has been linked to the release of pro-inflammatory cytokines, such as IL-6, IL-8, GM-CSF, and TNF [160,161]. As a result of these pro-inflammatory cytokines, BCG boasts a robust innate immune response with the recruitment of neutrophils and macrophages [159,162]. When BCG peptides are presented on MHC II of APCs, T helper cells are polarized to the Th1 type [163,164]. These Th1 cells are then able to support CD8^+^ T cells in their cytotoxic response against BC cells mediated via interaction with MHC I [165]. Lombardo et al. recently showed that BCG activates the cGAS-STING pathway, leading to both polarization of macrophages to the pro-inflammatory M1 type, and high ratios of Teff to Treg cells, forming a potent immune response [166].

Besides triggering innate and adaptive immunity, BCG also displays direct cytotoxic effects on BC cells, including activation of the caspase 8 signaling pathway after binding to TLR7 [167]. Another study implicated BCG in the activation of pro-apoptotic protein BID and pro-caspase 9, which eventually results in apoptosis [168]. Elevation of the HMGB1 protein, a marker for necrosis, also suggested that BCG treatment can lead to direct induction of necrosis in urothelial carcinoma cells [169,170]. A big drawback of a lot of these studies is the lack of in vivo evidence, which means that any of these discoveries could possibly be cell line dependent. Further studies are required to confirm the mechanisms by which BCG induces direct cytotoxicity in urothelial carcinoma. Altogether, effective BCG therapy remodels the bladder TME to exhibit pro-inflammatory characteristics through the release of pro-inflammatory cytokines and the activation of both innate and adaptive immunity.

Although few studies demonstrate a correlation between microbiome composition at baseline and BCG response, the potential relevance of the urinary microbiome in BC treatment is becoming increasingly evident. Knorr et al. investigated the microbiome of patients prior to BCG treatment, and found no significant differences in microbiome composition richness between responders and non-responders, but did find an enrichment of *Corynebacterium* and *Pseudomonas* among BCG responders [171]. This study implies that *Corynebacterium* could be used as a positive marker because it shares taxonomic similarities with *M. bovis* [171]. Sweis et al. reported that BCG-treated patients with enriched *Firmicutes*, such as *Lactobacillus*, did not experience recurrence [172]. However, Proteobacteria levels were significantly higher among patients with recurrences following BCG therapy [172]. As numerous types of bacteria including BCG can adhere to the extracellular matrix of the bladder mucosa, it is conceivable for urinary microbiota to saturate the BCG-binding sites [5]. The specific composition of urinary microbiota may diminish BCG efficacy and downregulate the robust cytotoxic response necessary to eliminate tumor cells [5]. In contrast, other types of microbiota (i.e., *Lactobacilli*) may generate antiproliferative and cytotoxic effects, thereby contributing to BCG’s antitumor activity and providing a synergistic effect [5,173]. Therefore, it needs a more detailed study design and precise microbiome analysis in this field. Recently, a larger sample size of BCG patients analyzed using 16S next-generation sequencing (NGS) and shotgun metagenomics revealed an enrichment of *Lactobacillus* spp. in BCG responders and an increase in *Corynebacterium* in BCG non-responders [174]. The increased BCG uptake of urothelial cancer carcinoma cells mediated by *Lactobacillus crispatus* could be a potential explanation for the BCG responsiveness of patients [174]. Additionally, as of May 2022, recruitment for clinical research to predict BCG responsiveness based on changes in the urinary microbiota is underway [175]. Hopefully, additional large-scale clinical investigations will offer information on the relationship between urinary microbiome composition and BCG response, with the ultimate objective of discovering clinical markers for formulating individually targeted treatment plans. Predictive markers that could exclude non-responding patients and allow physicians to only administer treatment to BCG-responsive patients could not only greatly reduce adverse effects, but also encourage more active research into alternative treatment methods for those patients who are not responsive to BCG.

## 8. Urinary Microbiome as Bladder Cancer Therapy

Not only can the composition of the urinary microbiome impact BCG treatment efficacy, but specific bacteria can also be employed as therapeutics. Takahashi et al. reported that *Lactobacillus casei* Shirota exhibited more potent anticancer effects than BCG in an orthotopic bladder tumor implantation mouse model, suggesting the role of beneficial bacterial species in treating human BC [15]. Comparing the efficacy of lyophilized *Lactobacillus rhamnosus* GG (LGG) preparations to BCG immunotherapy in a murine orthotopic tumor model revealed that the ability of LGG preparations to treat BC in mice was comparable to that of BCG immunotherapy [16]. *Lactobacillus rhamnosus* GG (LGG) and *Lactobacillus casei* Shirota were reported to kill BC cells through apoptosis and necrosis [173]. Additionally, *Lactobacillus acidophilus* was discovered to have a synergistic impact with BCG in the therapy of EJ138 bladder cancer cells via the upregulation of TNF-α cytokine and apoptotic *Bax* gene expression [176]. In addition, the immunomodulatory effects of LGG also promoted tumor regression by increasing the expression of lymphotactin (XCL1) to recruit T and NK cells [16,177,178]. Intravesical instillation of LGG induced the recruitment of activated DCs and an increase in the number of macrophages and neutrophils in the bladder. This study also demonstrated that LGG induces greater TNF-α production in neutrophils than BCG, which may have a positive effect on tumor regression [16]. Another approach to utilizing *Lactobacillus* species in treating BC is oral administration combined with intravesical immunotherapy. Naito et al. observed a higher 3-year recurrence-free survival rate for TURBT patients who received *Lactobacillus casei* orally in addition to an intravesical instillation of epirubicin compared to the group treated with epirubicin alone [17]. Increasingly, the application of *Lactobacilli* via direct intravesical instillation or oral administration with epirubicin therapy confirms the effectiveness of BC treatment. In contrast to the safety concerns surrounding BCG, *Lactobacilli* are regarded as safe and are utilized as a probiotic supplement. Therefore, *Lactobacilli* may be a safer and more effective option than immunotherapy for BC.

## 9. Microbiome and Immune Checkpoint Immunotherapy

Cytotoxic T-lymphocyte antigen 4 (CTLA-4) is a member of the CD28 family; it binds co-stimulatory B7 molecules with a higher affinity than CD28 and causes a negative feedback loop in the early T cell response [179]. Programmed cell death protein 1 (PD-1) is another co-inhibitory receptor that is present on the surface of numerous subtypes of tumor-infiltrating leukocytes [180]. Its ligands, PD-L1, are predominantly expressed on numerous cancer cell types, including BC cells. As a result of their anti-tumor immunity, inhibiting the interaction between CTLA-4 and B7 ligands or blocking the interaction between PD-1 and PD-L1 showed great success. Ipilimumab (CTLA-4 inhibitor) was evaluated in a clinical trial to determine the efficacy of combining Ipilimumab with conventional chemotherapeutics (Gemcitabine and Cisplatin) as a cancer immunotherapy for metastatic urothelial carcinoma patients [181]. The effectiveness of this strategy was validated; however, optimal combination adjustments were required [181]. Atezolizumab (PD-L1 inhibitor) was clinically shown to be successful in BC patients with locally progressed or metastatic urothelial carcinoma [182]. Nivolumab (PD-1 blockade drug) is also an effective treatment for patients with locally advanced or metastatic urothelial carcinoma [183].

The significance of the microbiota in bladder cancer may extend beyond NMIBC to include more advanced disease stages. Utilization of immunotherapy drugs, particularly those employing the PD-1/PD-L1 axis, has increased in early-stage and metastatic urothelial carcinoma [56,57]. Microbiota is one of the components having mechanisms involved in PD-L1 expression regulation [184,185]. Thereby, the bladder cancer response to anti-PD-1/PDL1 therapy may be associated with particular microbial compositions of the bladder microbiome. In certain epithelial malignancies, microbiota can induce immune evasion or immunological tolerance by upregulating the expression of PD-L1. Dysbiosis in oral mucosa upregulated PD-L1 in squamous carcinoma cells and gingival keratinocytes, and thus may support immune evasion of oral carcinomas [186]. Although this has not been examined systematically in bladder cancer, a significant correlation exists between the presence of *Bifidobacterium longum*, *Collinsella aerofaciens*, and *Enterococcus faecium* and a favorable response to therapy for metastatic melanoma [187]. Long-standing research has examined the function of PD-L1/PD1-mediated immune evasion in BC recurrence and progression [188,189]. Patients with positive PD-L1 expression have greater NMIBC recurrence and progression rates than those with negative PD-L1 expression. Comparative analysis of the urogenital microbiota between PD-L1-positive and PD-L1-negative groups was conducted using 16S rRNA gene sequencing. In the PD-L1-positive group, certain bacterial taxa (e.g., *Leptotrichia*, *Roseomonas*, and *Propionibacterium*) were enriched, whereas others (e.g., *Prevotella* and *Massilia*) were decreased. Studies have implicated *Leptotrichia* in the development of cancers such as gastric cancer, colon cancer, pancreatic cancer, and others [190,191,192]. This investigation demonstrated that these genera may influence the expression of PD-L1, although the precise mechanism remains unknown [193]. 

Lastly, the use of immune checkpoint inhibitors in cancer treatment has been expanded to include neoadjuvant immunotherapy in order to improve the efficacy of MIBC treatment and is producing positive clinical trial results [194,195]. However, no evidence exists demonstrating a correlation between urinary microbiota and the efficacy of neoadjuvant immunotherapy. Recently, Rajji et al. reported that antibiotics were related to diminished bladder cancer neoadjuvant immunotherapy benefit [196]. Based on this study, we can speculate that the diversity and composition of urinary microbiota may be related to the success of neoadjuvant immunotherapy. Future research is necessary to define the interaction between the urinary microbiota and these innovative immunotherapies.

## 10. Conclusions

The global prevalence of BC has generated widespread alarm, and the role of chronic inflammation in the development of BC is becoming increasingly evident. Efforts are ongoing to study the influence of microbiota on chronic inflammation regulated by the immune response to increase understanding of BC etiology. Characterizing the link between the urinary microbiome and chronic inflammation in the bladder may be essential to develop BC preventive and treatment strategies. The manipulation of the urinary microbiome to modify the inflammatory microenvironment will be crucial for improving the prognosis of BC patients. Future microbiome research translation into clinical practice will require the multidisciplinary integration of microbiome, immunity, and tumorigenesis for the successful development of cancer therapy.

## Figures and Tables

**Table 1:** Urinary microbiome in a healthy human bladder.

Type of BC	Enriched Genera	Sex	Urine Collection	Reference
Healthy subjects only	*Staphylococcus, Streptococcus, Lactobacillus*	F	Midstream urine	Curtiss et al. [33]
Healthy subjects only	Genera of the phyla *Firmicutes*, *Actinobacteria*, *Bacteroidetes*	F, M	Midstream urine	Lewis et al. [9]
Healthy subjects only	*Lactobacillus, Streptococcus*, *Gardnerella, Escherichia*	F	Transurethral catheter	Price et al. [32]
Healthy subjects only	M: *Enterococcus, Proteus*, *Klebsiella* F: *Lactobacillus*	F, M	Midstream urine, transurethral catheter	Fouts et al. [10]
Healthy subjects only	*Lactobacillus*, *Prevotella*, *Garnderella*	F	Midstream urine	Siddiqui et al. [29]
Healthy subjects only	*Lactobacillus*, *Staphylococcus*	F	Transurethral catheter	Pearce et al. [30]
Healthy subjects only	*Lactobacillus, Gardnerella, Gardnerella/Prevotella*	F	Transurethral catheter	Pearce et al. [34]
Healthy subjects only	*Lactobacillus*, *Gardnerella*, *Corynebacterium*	F	Midstream urine, transurethral catheter	Thomas-White et al. [35]
Study on microbiome in men with kidney stones	*Prevotella*, *Lactobacillus*	M	Transurethral catheter	Xie et al. [36]

**Table 2:** Urinary microbiome in bladder cancer patients.

Type of BC	Enriched Genera	Sex	Description	Urine Collection	Reference
Mixed	*Fusobacterium, Actinobaculum*, *Facklamia,* *Campylobacter*	M	No difference in alpha diversity	Midstream urine	Bucevic Popovic et al. [8]
Mixed	*Acinetobacter*, *Anaerococcus*, *Rubrobacter*	M	Increased bacterial richness in cancer, significant beta diversity	Midstream urine	Wu et al. [14]
Recurrent NMIBC	*Staphylococcus*, *Streptococcus*, *Prevotella*	M	Increased bacterial richness in cancer, no differences in Shannon and Simpson indices, species diversity higher in recurrence group, more OTUs in cancer	Midstream urine	Zeng et al. [39]
MIBC, NMIBC	*Bacteroides*, *Akkermansia*, *Klebsiella*	M, F	No relationship found between microbiota in NMIBC and MIBC	Transurethral resection (tissue)	Mansour et al. [23]
MIBC, NMIBC	*Lactobacillus*, *Corynebacterium*, *Streptococcus*	M, F	No relationship found between microbiota in NMIBC and MIBC	Transurethral resectoscopy (urine)	Mansour et al. [23]
MIBC	*Bacteroides*, *Faecalibacterium*	M, F	Decreased bacterial richness in cancer	Midstream urine	Chipollini et al. [40]
Mixed	*Brucellaceae, Acinetobacter*, *E.Shigella*, *Proteobacteria*	M, F	Study on noncancerous vs. cancerous tissue;lower Shannon diversity in cancerous tissue, lower degrees of species richness and diversity in cancer	Tissue collected intraoperatively	Liu et al. [22]
Mixed	*Actinomyces, Achromobacter*, *Brevibacterium* phyla: *Actinobacteria*, *Proteobacteria*	M, F	No difference in alpha diversity but higher beta diversity	Midstream urine, Transurethral catheter	Hussein et al. [41]
Mixed	*Veillonella*, *Corynebacterium*	M	Significantly higher evenness parameter in BC group	Transurethral catheter	Oresta et al. [42]
